# What is an appropriate gestational weight gain for women with gestational diabetes mellitus: based on the adverse pregnancy outcomes of over 12 thousand participants?

**DOI:** 10.1186/s13098-022-00940-8

**Published:** 2022-11-11

**Authors:** Xiaoqin Luo, Jiayi Gao, Zhangya He, Jing Ji, Wanyu Zhang, Pei Wu, Xiaoxiao Guo, Dan Cao, Zhangrui Xu, Chao Li, Yang Mi

**Affiliations:** 1 Department of Nutrition and Food Safety, School of Public Health, Xi’an Jiaotong University, Xi’an, 710061 China; 2 Emergency Medical Center, Xi’an Public Health Center, Xi’an, 710200 China; 3 Department of Obstetrics and Gynecology, Northwest Women and Children’s Hospital, Xi’an, 710061 China; 4 Shaanxi Health Supervision Center, Xi’an, 710077 China; 5 Department of Epidemiology and Health Statistics, School of Public Health, Xi’an Jiaotong University, Xi’an, 710061 China

**Keywords:** Gestational diabetes mellitus, Gestational weight gain, Pregnancy, Pregnancy outcomes

## Abstract

**Background:**

Evidence showed possible benefits of a less gestational weight gain (GWG) than the US Institute of Medicine (IOM) recommendation in gestational diabetes mellitus (GDM) pregnancy. Here, we aimed to explore an appropriate GWG range in GDM women according to adverse pregnancy outcomes.

**Methods:**

We enrolled all the singleton GDM pregnant women (n = 14,213) from January 2015 to December 2018 in Xi'an, Northwest China. According to the pre-pregnancy body mass index (BMI), they were classified into the Underweight (< 18.5 kg/m^2^), Normal weight (18.5–24.9 kg/m^2)^, Overweight (25.0–29.9 kg/m^2^) and Obesity (≥ 30.0 kg/m^2^) group, respectively. Logistic regression analysis was used to calculate the odds ratio (OR) and 95% confidence intervals (95% CI). The appropriate ranges of GWG were determined based on a significant protective association (OR < 1).

**Results:**

Totally, 12,712 participants were finally recruited. There were 1180 (9.3%), 9134 (71.9%), 2097 (16.5%), and 301 (2.4%) patients in the Underweight, Normal weight, Overweight, and Obesity groups, respectively. Adverse outcomes increased with the elevation of pre-pregnancy BMI. Among them, the risk of cesarean section was the highest, followed by large for gestational age (LGA), small for gestational age (SGA), preeclampsia, and gestational hypertension. Through the analysis of the risk of adverse outcomes in continuous GWG categories in each group, an ideal GWG range obtained in this study was as follows: 10–15.9 kg, 8–11.9 kg, 6–7.9 kg, and -5–3.9 kg for the Underweight, Normal weight, Overweight and Obesity group, respectively. Furthermore, the ranges in this study were more protective for adverse outcomes than those from IOM.

**Conclusions:**

Based on the adverse pregnancy outcomes of over 12 thousand participants, our findings showed a more stringent GWG range for GDM women than the IOM criteria recommendation.

**Supplementary Information:**

The online version contains supplementary material available at 10.1186/s13098-022-00940-8.

## Background

Gestational diabetes mellitus (GDM) is diagnosed during the 2nd or 3rd trimester of pregnancy that is not either preexisting type 1 or type 2 diabetes [1]. A meta-analysis involving 79,064 Chinese participants showed that the total incidence of GDM in mainland China was 14.8% (95% confidence interval of 12.8–16.7%) [2]. GDM has been reported to be associated with a lot of adverse pregnancy outcomes, such as large for gestational age (LGA), macrosomia, shoulder dystocia, cesarean section, pregnancy-induced hypertension, and pre-eclampsia [3, 4]. Long-termly, GDM may increase the risk for glucose metabolism disorders and dyslipidemia after pregnancy in mothers, and childhood obesity, insulin resistance, and atherosclerotic lipid properties in offspring [5, 6].

Excessive gestational weight gain (GWG) usually leads to both short-term and long-term adverse pregnancy outcomes directly itself and indirectly as a mediator of GDM [7]. Studies have shown that women with excessive GWG are more likely to have abdominal obesity and increased metabolic diseases [8, 9]. With the implementation of China's comprehensive two-child policy, the weight retention caused by excessive GWG will be another important fuse for a new round of metabolic diseases in pregnancy [10]. In addition to affecting the mothers, GWG is also an independent predictor of obesity and total body fat distribution in infancy offspring and affects offspring’s cardiovascular metabolism in their adulthood [11, 12]. Besides, insufficient GWG also doesn’t benefit the offspring by increasing the risk of premature delivery, small gestational age (SGA), and low birth weight [13, 14].

Therefore, an appropriate GWG suggestion is urgently needed for women with GDM. Unfortunately, there are currently no specific guidelines in any country, including China. In China, the clinical guidelines revised by the US Institute of Medicine (IOM) in 2009 [15], recommended GWG for all pregnant women according to pre-pregnancy body mass index (BMI) is still being used. Not to mention in women with GDM, such a recommendation is even inappropriate for pregnant women with normal glucose metabolism [16]. A previous study has shown that in GDM pregnancy, GWG less than the recommended weight will be beneficial [17]. However, the ideal range of GWG has not been determined. Therefore, using the data from the Xi’an longitudinal mother–child cohort (XAMC) study, we explored the appropriate ranges of GWG for GDM women with different pre-pregnancy BMI categories.

## Methods

### Population and data sources

The present analysis is based on data from the XAMC study, which was established in January 2013 and the enrollment will be expected to end in January 2023. The research protocol and basic information were previously published [18]. Based on the dynamically conducted XAMC study, we enrolled all the singleton GDM pregnant women (14,213) from January 2015 to December 2018 from Northwest Women’s and Children’s Hospital, Xi’an, Northwestern of China. The inclusion criteria were as follows: women who were diagnosed with GDM by receiving a 75 g oral glucose tolerance test (OGTT) between 24 and 28 weeks of gestation according to criteria of the International Association of Diabetes in Pregnancy Study Groups (IADPSG) (75 g OGTT fasting blood glucose  ≥ 5.1 mmol/L, or 1 h blood glucose  ≥ 10.0 mmol/L, or 2 h blood glucose  ≥ 8.5 mmol/L) [19]; full-term singleton pregnancy (gestational age  ≥ 37 weeks); completed data including height, weight, GWG, maternal and fetal outcomes and so on. The exclusion criteria were non-gestational diabetes or diabetes diagnosed before pregnancy; multiple births; premature delivery; abortion or induced labor; incomplete or incorrect data. The protocol was approved by the ethical committee of Xi’an Jiaotong University (XJTU 2016-053) and the Northwest Women and Children’s Hospital (NWCH 2012-013). All women provided gave written informed consent. The principles of the Helsinki Declaration were followed throughout the study.

### Maternal pre-pregnancy BMI and GWG

When firstly diagnosed being pregnant (usually before 6 gestational weeks), women’s maternity booklet was created at hospital and information of height and weight were measured by a professional medical staff and recorded as pre-pregnancy height and weight. Maternal pre-pregnancy BMI was calculated by dividing the pre-pregnancy weight by the square of the height. The gestational weight gain (GWG) was calculated by subtracting pre-pregnancy weight from the weight measured before delivery. The Underweight, Normal weight, Overweight and Obesity group were defined with pre-pregnancy BMI < 18.5 kg/m^2^, 18.5–24.9 kg/m^2^, 25.0–29.9 kg/m^2^ and ≥ 30.0 kg/m^2^, respectively.

### Pregnant outcomes

Adverse outcomes of this study were defined as the presence of at least one of the following outcomes: gestational hypertension, preeclampsia, cesarean delivery, and SGA or LGA. Gestational hypertension was defined as systolic blood pressure (SBP)  ≥ 140 mmHg and/or diastolic blood pressure (DBP)  ≥ 90 mmHg occurring for the first time after 20 weeks of pregnancy. Pre-eclampsia was defined as gestational hypertension plus proteinuria. LGA was defined as an infant whose birth weight was above the 90th percentile of the average birth weight of the same gestational age, whereas SGA was below the 10th percentile according to the INTERGROWTH-21st Project [20].

### Data quality control

In this study, two trained data collectors performed parallel data entry using the Epidata 3.1 software. When completed, a consistency check was performed. Other personnel with professional knowledge checked the outliers, which were defined when beyond 3 times the standard deviation of the average. Data judged as outliers were double-checked and set as missing values if it were not a data entry error. The missing value analysis is in Additional file 1: Table S1 and S2.

### Statistical analysis

Quantitative data were tested by analysis of variance or rank-sum test, and qualitative data were tested by *Chi-square*. The absolute risk was calculated as the percentage of women with adverse outcomes in each combination of the BMI and GWG categories. The Logistic regression analysis was used to calculate the odds ratio (OR) and 95% confidence intervals (95% *CI*) of adverse outcomes for each GWG range in a specific pre-pregnancy BMI group with the adjustment of age, parity, gestational week, and previous cesarean section history. The reference for each GWG category was the GWG beyond the range. The risk of adverse outcomes in continuous GWG categories in each group was analyzed. The appropriate GWG ranges were determined based on whether the GWG range was a protective factor for adverse outcomes (OR value < 1). According to the obtained GWG ranges or the IOM recommended ones (12.5–18.0 kg, 11.5–16.0 kg, 7.0–11.5 kg, and 5.0–9.0 kg for the Underweight [BMI < 18.5 kg/m^2^], the Normal weight [BMI 18.5–24.9 kg/m^2^], the Overweight [BMI 25.0–29.9 kg/m^2^] and the Obesity [BMI ≥ 30.0 kg/m^2^], respectively), the sensitivity, specificity, positive predictive value and negative predictive value were calculated. Furthermore, the Net Reclassification Index (NRI) was calculated to assess the prediction ability of GWG obtained in this study and the one from IOM. *P* < 0.05 was considered statistically significant.

## Results

### Basic characteristics of participants

As shown in Additional file 1: Figure S1, a total of 12,712 participants were finally enrolled in this study, of which 9.3% (1180) were in the Underweight group; 71.9% (9134) in the Normal weight group; 16.5% (2097) in the Overweight group and 2.4% (301) in the Obesity group (Table 1). Compared with the Normal weight group, the Underweight group was younger and the Overweight group was older. The proportion of participants with a family history of diabetes and hypertension in each group increased from the Underweight group to the Obesity group (8.1%, 9.6%, 11.5%, and 16.9%, respectively for diabetes while 10.4%, 14.7%, 17.6%, and 22.9%, respectively for hypertension). More participants had a history of previous cesarean section or adverse pregnancy in the Normal weight group and Overweight group than that in the Underweight group whilst the situation was the same as multiparous. The majority of basic characteristics were comparable between the Obese group and the Overweight group.

**Table 1:** Characteristics of participants in different BMI categories

Characteristic	Pre-pregnancy Body Mass Index				*P-value*^a^
	Underweight (BMI < 18.5)	Normal weight (BMI18.5 ~ 24.9)	Overweight (BMI25.0 ~ 29.9)	Obesity (BMI ≥ 30.0)	
N	1180 (9.3)	9134 (71.9)	2097 (16.5)	301 (2.4)	
Age(years)	30 (28–32)^b^	31 (29–34)^c^	32 (29–35)^d^	31 (29–34)^cd^	< 0.001
< 35	1020 (86.4)^b^	6852 (75.0)^c^	1501 (71.6)^d^	232 (77.1)^cd^	< 0.001
≥ 35	160 (13.6)^b^	2282 (25.0)^c^	596 (28.4)^d^	69 (22.9)^cd^	
Height(cm)	162.0 (160.0–165.0)^b^	162.0 (159.0–165.0)^c^	162.0 (159.0–165.0)^c^	162.0 (160.0–165.0)^bc^	< 0.001
Pre-pregnancy Weight(kg)	46.0 (44.0–49.0)^b^	56.5 (52.5–60.0)^c^	70.0 (66.0–74.0)^d^	84.0 (79.5–90.0)^e^	< 0.001
Pre-pregnancy BMI(kg/m^2^)	17.7 (17.0–18.1)^b^	21.6 (20.2–23.0)^c^	26.4 (25.6–27.6)^d^	31.4 (30.5–33.3)^e^	< 0.001
Education level					< 0.001
Low	140 (11.9)^b^	1204 (13.2)^b^	365 (17.4)^c^	64 (21.3)^c^	
Medium	832 (70.5)^b^	6387 (69.9)^b^	1473 (70.2)^b^	214 (71.1)^b^	
High	194 (16.4)^b^	1415 (15.5)^b^	231 (11.0)^c^	20 (6.6)^c^	
Missing	14 (1.2)^b^	128 (1.4)^b^	28 (1.3)^b^	3 (1.0)^b^	
Oral glucose tolerance test					
Fasting plasma glucose level(mmol/L)	5.11 (4.66–5.30)^b^	5.19 (4.88–5.40)^c^	5.29 (5.10–5.56)^d^	5.32 (5.13–5.58)^d^	< 0.001
1 h plasma glucose level(mmol/L)	9.05 ± 1.78^b^	9.24 ± 1.73^c^	9.64 ± 1.80^d^	9.79 ± 1.69^d^	< 0.001
2 h plasma glucose level(mmol/L)	7.89 ± 1.48^b^	7.96 ± 1.48^b^	8.07 ± 1.52^c^	7.78 ± 1.50^b^	0.001
History of adverse pregnancy	473 (40.1)^b^	4495 (49.2)^c^	1092 (52.1)^c^	154 (51.2)^c^	< 0.001
History of previous cesarean section	131 (11.1)^b^	1550 (17.0)^c^	474 (22.6)^d^	65 (21.6)^cd^	< 0.001
Parity					< 0.001
Nulliparous	859 (72.8)^b^	5583 (61.1)^c^	1207 (57.6)^d^	180 (59.8)^cd^	
Multiparous	321 (27.2)^b^	3551 (38.9)^c^	890 (42.4)^d^	121 (40.2)^cd^	
Family history of diabetes	95 (8.1)^b^	875 (9.6)^b^	241 (11.5)^c^	51 (16.9)^d^	< 0.001
Family history of hypertension	123 (10.4)^b^	1342 (14.7)^c^	370 (17.6)^d^	69 (22.9)^d^	< 0.001
Delivery					
Weight(kg)	61.0 (57.0–65.0)^b^	70.0 (66.0–75.0)^c^	81.6 (77.0–87.0)^d^	94.0 (88.0–100.0)^e^	< 0.001
Total GWG (kg)	15.0 (12.0–18.0)^b^	14.0 (11.0–17.0)^c^	12.0 (9.0–15.0)^d^	10.0 (7.0–14.0)^e^	< 0.001
Gestational age (weeks)	39.0 ± 1.02^bc^	39.0 ± 1.03^c^	38.9 ± 1.04^b^	38.9 ± 1.11^b^	< 0.001
Birth weight (g)	3250 (3012–3500)^b^	3415 (3150–3690)^c^	3500 (3210–3800)^d^	3560 (3210–3900)^d^	< 0.001
Biochemical indicators at the end of pregnancy					
HbA1c (%)	5.2 (5.0–5.5)^b^	5.3 (5.1–5.6)^c^	5.4 (5.1–5.7)^d^	5.5 (5.3–5.8)^e^	< 0.001
HbA1c (mmol/mol)	33 (31–37)	34 (32–38)	36 (32–39)	37 (34–40)	
Total cholesterol(mmol/L)	5.89 ± 1.15^b^	5.74 ± 1.18^c^	5.52 ± 1.13^d^	5.28 ± 1.14^e^	< 0.001
HDL(mmol/L)	1.75 ± 0.43^b^	1.71 ± 0.41^c^	1.65 ± 0.37^d^	1.61 ± 0.34^d^	< 0.001
LDL(mmol/L)	3.06 ± 0.74^b^	2.87 ± 0.75^c^	2.74 ± 0.73^d^	2.63 ± 0.73^d^	< 0.001
Triglycerides(mmol/L)	2.68 (2.14–3.45)^b^	3.02 (2.37–3.91)^c^	3.10 (2.45–4.01)^c^	3.12 (2.51–3.93)^c^	< 0.001
Adverse outcomes	467 (39.6)^b^	5160 (56.5)^c^	1521 (72.5)^d^	241 (80.1)^e^	< 0.001
Gestational hypertension	10 (0.8)^b^	149 (1.6)^b^	91 (4.3)^c^	31 (10.3)^d^	< 0.001
Preeclampsia	10 (0.8)^b^	173 (1.9)^b^	107 (5.1)^c^	36 (12.0)^d^	< 0.001
Cesarean section	369 (31.3)^b^	4128 (45.2)^c^	1257 (59.9)^d^	204 (67.8)^d^	< 0.001
LGA	123 (10.4)^b^	1889 (20.7)^c^	645 (30.8)^d^	110 (36.5)^d^	< 0.001
SGA	39 (3.3)^b^	224 (2.5)^b^	40 (1.9)^b^	4 (1.3)^b^	0.049

Categorical variables are expressed as counts (percentages) and the chi-square test is used. If the number of cases is less than 40, or the number of grids with an expected value of less than 5 > 25% of the total number of grids, then Fisher’s exact probability test is used

*HDL* High Density Lipoprotein, *LDL* Low Density Lipoprotein, *LGA* Large for Gestational Age, *SGA* Small for Gestational Age

^a^Continuous variable that meets the conditions of normality and homogeneity of variance is expressed as mean ± standard deviation, using analysis of variance, and LSD is used for pairwise comparison between groups; otherwise, a non-parametric test is used and expressed as the median (quartile)

^bcde^There is no significant difference between groups with the same symbol

*P* < 0.05 were considered statistically significant

### The GWG and the indicators of glucose metabolism

The median total GWG decreased with pre-pregnancy BMI, which was 15.0 (12.0–18.0) kg, 14.0 (11.0–17.0) kg, 12.0 (9.0–15.0) kg, and 10.0 (7.0–14.0) kg for the Underweight, Normal weight, Overweight and Obesity group, respectively. The OGTT data showed that the blood glucose level also increased with pre-pregnancy BMI. In detail, the fasting blood glucose was 5.11, 5.19, 5.29, and 5.29 mmol/L, the 1 h blood glucose was 9.05, 9.24, 9.64, and 9.79 mmol/L, while the 2 h blood glucose was 7.89, 7.96, 8.07 and 7.78 mmol/L in the Underweight, Normal weight, Overweight and Obesity group, respectively. There was no significant difference in OGTT level between the Overweight group and the Obesity group. Meanwhile, the late pregnancy HbA1c levels increased with pre-pregnancy BMI as well. The birth weight of the fetus in the Normal weight group (3415 [3150–3690] g) was higher than the Underweight group (3250 [3012–3500] g) but smaller than the Overweight group (3500 [3210–3800] g) and Obesity group (3560 [3210–3900] g). No significant difference in birth weight between the Overweight group and the Obesity group was reported (Table 1).

### Incidence of adverse pregnancy outcomes

**Fig. 1:**
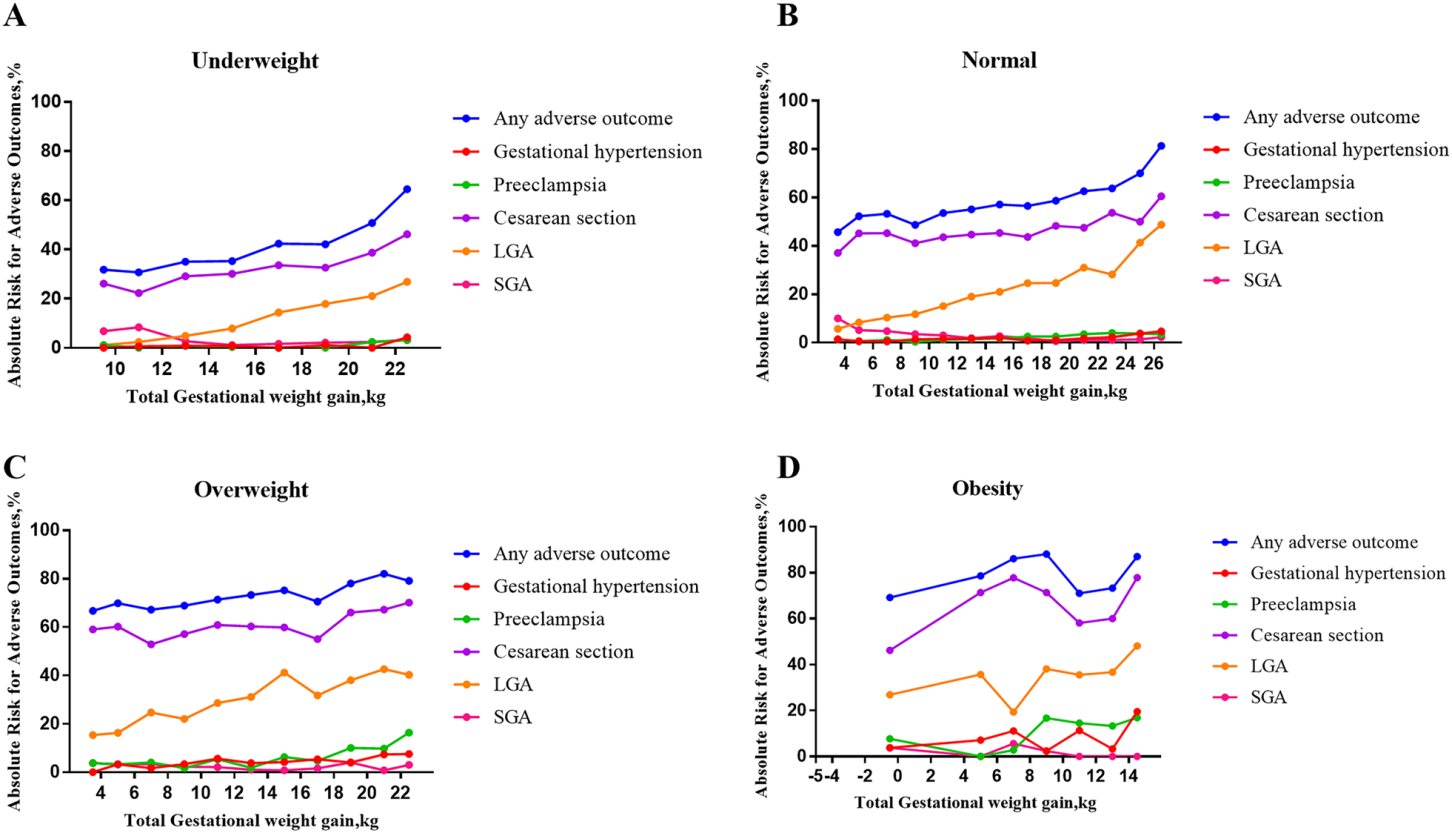
Absolute Risk for Adverse Maternal or Infant Outcomes. Absolute risk = (No. of participants with the adverse outcomes/No. of participants in GWG category within pre-pregnancy BMI group) × 100. The total GWG was divided by the group distance of 2 kg, except for the first GWG range and the last GWG range of each pre-pregnancy BMI group

Overall, adverse outcomes increased with pre-pregnancy BMI, which were 39.6%, 56.5%, 72.5%, and 80.1% in the Underweight, Normal weight, Overweight, and Obesity groups, respectively. The incidence of gestational hypertension and preeclampsia increased sequentially both from 0.8% in the Underweight group to 10.3% and 12.0% in the Obesity group, respectively. The cesarean section rate of the Normal weight group (45.2%) was higher than that of the Underweight group (31.3%) but lower than that of the Overweight group (59.9%) and Obesity group (67.8%). In addition, the Obesity group had the highest risk of LGA (36.5%), and the Underweight group was the lowest (10.4%). No significant difference was found in the incidence of cesarean section and LGA between the Overweight and the Obesity group. Although the incidence of SGA among the four groups was comparable, it decreased gradually with pre-pregnancy BMI (Table 1). Generally, the absolute risk of adverse outcomes increased with the increase of GWG in all groups (Fig. 1). Among all the adverse outcomes mentioned in this study, the risk of cesarean section was the highest, followed by LGA, SGA, preeclampsia, and gestational hypertension. Interestingly, in the Underweight and the Normal weight group, the risk of SGA was higher than that of LGA only when the GWG was lower. This phenomenon occurred when the GWG was less than 12 kg in the Underweight group and 4 kg in the Normal weight group. However, the risk of LGA was always higher than that of SGA in the other two groups (Additional file 1: Table S3).

### The appropriate GWG range and its verification

**Fig. 2:**
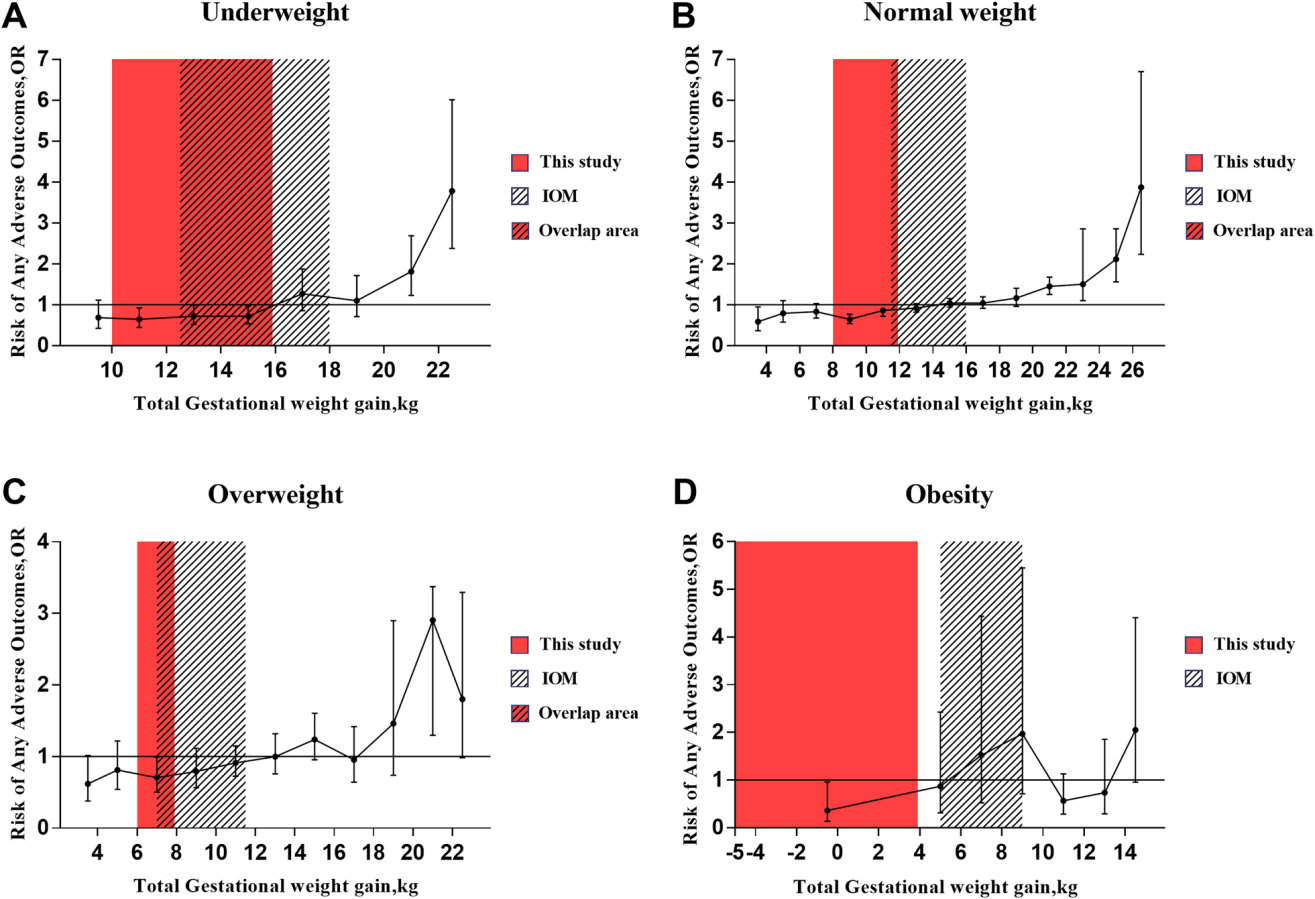
Associations of GWG Categories with Adverse Outcomes. The filled circles and error bars indicate OR and 95% *CI*, respectively. The red area represents the optimal GWG range according to this study, the area with black slant lines represents the GWG ranges recommended by IOM. The overlapped area is shown in red with black slant lines. The total GWG was divided by the group distance of 2 kg, except for the first GWG ring and the last GWG range of each pre-pregnancy BMI group. The GWG category with a protective association was defined as the optimal GWG range. Combining the results of the four groups of models, the results of Model 3 are used for the Underweight group, the results of Model 1 are used for the Normal weight group and Overweight group, and the results of Model 2 are used for the Obesity group

As shown in Additional file 1: Table S4, the risk of adverse outcomes increased by 1.153 (95% *CI* 1.139–1.167) and 1.021 (95% *CI* 1.014–1.029) for each increase of 1 unit of pre-pregnancy BMI and GWG, respectively. We next analyzed the risk of adverse outcomes in continuous GWG categories in each group (Fig. 2). The GWG category with a protective association was defined as the appropriate GWG range. As shown in Additional file 1: Table S5–S8, the appropriate GWG range was 10–15.9 kg, 8–11.9 kg, 6–7.9 kg, and -5–3.9 kg for the Underweight, Normal weight, Overweight, and the Obesity group, respectively.

As shown in Table 2, the sensitivity of the optimal GWG range obtained in this study was all higher than that recommended by IOM, while the specificity was the opposite. Except for SGA, positive and negative predictive values were higher than those of IOM. To compare the accuracy of the prediction capabilities of the two GWG recommended ranges, the Net Reclassification Index (NRI) was calculated. The NRI value was 0.065 > 0, indicating that the predictive ability of GWG recommended ranges in this study was better compared with the ones from IOM (Additional file 1: Table S9).

**Table 2:** Sensitivity, specificity, positive predictable value and negative predictable value in the recommended GWG range of IOM and this study

	Recommended GWG range of this study				Recommended GWG range of IOM			
	Sensitivity (%)	Specificity (%)	Positive predictable value (%)	Negative predictable value (%)	Sensitivity (%)	Specificity (%)	Positive predictable value (%)	Negative predictable value (%)
Adverse outcomes	79.6	29.0	60.9	47.3	57.9	44.7	59.2	43.3
Gestational hypertension	84.7	24.2	2.5	98.6	59.8	43.2	2.3	97.9
Preeclampsia	89.9	24.3	3.0	98.9	63.5	43.3	2.9	97.8
Cesarean section	79.4	26.9	48.9	59.7	57.7	44.0	47.6	54.1
LGA	85.6	26.6	24.5	86.9	59.6	43.9	22.8	79.6
SGA	67.8	23.8	2.2	96.8	59.9	43.2	2.5	97.8

*LGA* Large for Gestational Age, *SGA* Small for Gestational Age

## Discussion

An appropriate GWG is crucial to reducing the risk of adverse pregnancy outcomes for GDM women and their offspring. Here, using the data from the XAMC cohort, we explored GWG ranges for GDM women with different pre-pregnancy BMI, which are stricter than the IOM recommendation. In detail, the ideal GWG range was 10–15.9 kg, 8–11.9 kg, 6–7.9 kg, and -5–3.9 kg for the Underweight, Normal weight, Overweight, and Obesity group, respectively.

In this study, we found that not only the blood glucose and HbA1c, but also the incidence of adverse outcomes, gestational hypertension, pre-eclampsia, cesarean section, and LGA raised with the increase in pre-pregnancy BMI. Nevertheless, pre-pregnancy BMI cannot be changed for pregnant women who have already been diagnosed with GDM. Furthermore, even in women of normal weight before pregnancy, excessive GWG will also have a moderate long-term impact on the cardiometabolic risk factors of adult offspring [12]. On the contrary, GWG is modifiable, and it still had a positive impact on the fetus even with the intervention conducted in the third trimester of pregnancy. Moreover, an appropriate GWG can attenuate the influence of pre-pregnancy BMI on childhood obesity [21]. Currently, the optimal GWG recommended by IOM is the most authoritative and widely used for all pregnant women. Accordingly, GDM women who had insufficient GWG were found to be at the lowest risk of adverse outcomes compared to those who had appropriate or excessive GWG [22]. A stricter GWG recommendation is needed for women with GDM [17, 23]. Consistently, our findings showed that, for GDM patients, the GWG ranges of each pre-pregnancy BMI category were stricter and lower than those recommended by IOM. Notably, among all participants included in the study, nearly one in five were overweight or obese before conception. In our study, the appropriate GWG range of the Obesity group was less than 4 kg and the lower limit even is -5 kg, indicating that weight loss during pregnancy in obese women with GDM may reduce adverse pregnancy outcomes.

Among all adverse pregnancy outcomes, cesarean section was the one with the highest incidence. A prospective birth cohort study in Southwest China showed that controlling GWG can reduce the incidence of cesarean section, and the optimal level of GWG in reducing the rate of cesarean section is more stringent than the IOM recommendation [24]. Importantly, we also observed a similar phenomenon in GDM women. When the GWG in each group was within the recommended ranges, the risk of cesarean section was significantly reduced, and more importantly, the risk was much lower in the ranges obtained in our study than in those from IOM. The situation of all other adverse outcomes was similar except for SGA. It has been found that a lower GWG will increase the risk of SGA [25]. Notably, since the GWG ranges obtained in our study were stricter, it significantly increased the risk of SGA. Considering LGA was the second major adverse outcomes with a remarkable high incidence than SGA in this study and the risk of LGA can be reduced within the recommended range of this study in all groups, the recommended range of GWG in our study should be more favorable for an appropriate birth weight if corresponding measures are taken to avoid the occurrence of SGA.

Clinically, when a pregnant woman comes to see a doctor for the first time, the doctor should recommend an appropriate GWG based on her pre-pregnancy BMI to reduce their risk of getting GDM and adverse pregnancy outcomes. If she is at a high risk of developing GDM, the doctor can directly advise her of an appropriate GWG during the entire pregnancy based on our recommendation. By the 24–28th week of pregnancy, if she is diagnosed with GDM, the doctor can conduct diet, exercise, and even insulin treatments, to control both blood sugar and weight gain. Although the ideal ranges we recommended are for the entire pregnancy, the doctor can also subtract the weight gain value before being diagnosed with GDM from them to roughly suggest the weight gain space for the patient in the later pregnancy.

GWG is necessary to ensure a healthy fetus, but excessive GWG, especially in women with GDM, has been associated with a high risk of adverse pregnancy outcomes. Women with different pre-pregnancy BMI have different GWG expectations. Existing guidelines for GWG from IOM have several key limitations [26], and it is not suitable for women with GDM. This study pooled GDM individual participant data from the XAMC study to explore optimal GWG ranges for women with different pre-pregnancy BMI. The ability of the optimal GWG range in this study to correctly predict adverse pregnancy outcomes in pregnant women with GDM is higher than that of IOM, and the GWG recommended range of IOM has a higher ability to correctly determine the absence of adverse pregnancy outcomes in pregnant women with GDM. As we described in the Clinical Implication, our findings will provide a chance for women with GDM to reduce the risk of getting adverse outcomes because of being suggested an appropriate GWG. However, whether women with GDM and those who are at high risk of being diagnosed with GDM can achieve the target GWG and whether this recommended GWG range can be promoted clinically remain to be further verified. This requires more effective and convenient interventions for GDM women to be developed. Notably, the ranges in our study were only based on the very short-term adverse pregnancy outcome. To make them more precise and have a greater guiding role in the clinical work, the long-term adverse outcomes are under observation with the follow-up of the XAMC study.

Our research had some limitations. Firstly, the timing and pattern of GWG will affect pregnancy outcomes [27, 28]. Consistent with the IOM guidelines, this study used total GWG to identify optimal GWG ranges instead of GWG per week because it does not have a linear pattern. Since this was a retrospective study, the dynamic changes of GWG were not obtained so it was impossible to evaluate how GWG patterns affected pregnancy outcomes in our study. Secondly, we only analyzed five important adverse outcomes (gestational hypertension, pre-eclampsia, cesarean section, LGA, and SGA) instead of all possible ones. This may lead to a possibility that the missing of some information may inevitably make this range suboptimal. Thirdly, information on the diet and physical activity of pregnant women was not available which may bring some bias. Fourth, although there is a big sample, the women are all from one single place in China and only a few adverse outcomes were evaluated, so the results cannot affirm that the proposed GWG rages can be used for all women with GDM around the world. A study of 1,309,136 pregnant women showed that the BMI of women from the United States and Europe was higher than that of Asia, and the GWG expectation was different [29]. Deputy et al. showed that in addition to the association between pre-pregnancy BMI and GWG, race-ethnicity is also related to GWG. Compared with white women of normal weight, the probability of insufficient GWG mainly occurs in blacks, Hispanics, and Asians with normal weight [30]. Besides, the loss of weight during pregnancy is a very controversial concern in the current research. Despite these limitations, our research also has several important strengths. Firstly, to the best of our knowledge, this is the first time appropriate GWG ranges are explored for women with GDM with different pre-pregnancy BMI categories. Secondly, the data obtained from such a large population made the results more convincing although they were only from a tertiary hospital in western China. Thirdly, many efforts were made to find optimal GWG in normal weight pregnant women and only a single outcome, such as birth weight or cesarean section was used [24, 31, 32]. Here, we analyzed adverse outcomes including five common ones to explore and assess the optimal range of GWG. Finally, a planned long-term follow-up based on the XAMC cohort is ongoing, which will verify the clinical value of this range and provide more valuable information for the exploration of a more suitable GWG for pregnant women with GDM.

## Conclusions

In conclusion, based on the adverse pregnancy outcomes of over 12 thousand participants in northwest of China, our findings showed a more stringent GWG range for GDM women than the IOM criteria recommendation.

## Supplementary Information

**Additional file 1: Figure S1. **Flow chart of the participants. **Table S1.** Description of Missing value. **Table S2.** *Chi*-square test for missing values. **Table S3.** Description of outcomes by GWG category. **Table S4.** Univariate logistic regression analysis of continuous GWG and pre-pregnancy BMI with adverse outcomes. **Table S5.** Associations of GWG categories with adverse outcomes in the Underweight group*. **Table S6.** Associations of GWG categories with adverse outcomes in Normal weight group*. **Table S7.** Associations of GWG categories with adverse outcomes in Overweight group*. Table S8. Associations of GWG categories with adverse outcomes in Obesity group*. **Table S9.** The results of adverse outcomes predicted by the logistic regression models in Table 2.

## Data Availability

The datasets used and/or analyzed during the current study are available from the corresponding author on reasonable request.

## Abbreviations

GDM: Gestational diabetes mellitus
GWG: Gestational weight gain
IOM: Institute of medicine
CI: Confidence intervals
OR: Odds ratio
LGA: Large for gestational age
SGA: Small for gestational age
BMI: Body mass index
XAMC: Xi’an longitudinal mother–child cohort
OGTT: Oral glucose tolerance test
IADPSG: International Association of Diabetes in Pregnancy Study Groups
XJTU: Xi’an Jiaotong University
NWCH: Northwest Women and Children’s Hospital
SBP: Systolic blood pressure
DBP: Diastolic blood pressure
NRI: Net Reclassification Index
