# Supplementary material for: What is an appropriate gestational weight gain for women with gestational diabetes mellitus: based on the adverse pregnancy outcomes of over 12 thousand participants?
Source: Diabetol Metab Syndr. 2022 Nov 11;14:166. doi: 10.1186/s13098-022-00940-8 (PMC9652847; doi:10.1186/s13098-022-00940-8)
Supplement: Supplementary file 1 — Additional file 1: Figure S1. Flow chart of the participants. Table S1. Description of Missing value. Table S2. Chi-square test for missing values. Table S3. Description of outcomes by GWG category. Table S4. Univariate logistic regression analysis of continuous GWG and pre-pregnancy BMI with adverse outcomes. Table S5. Associations of GWG categories with adverse outcomes in the Underweight group*. Table S6. Associations of GWG categories with adverse outcomes in Normal weight group*. Table S7. Associations of GWG categories with adverse outcomes in Overweight group*. Table S8. Associations of GWG categories with adverse outcomes in Obesity group*. Table S9. The results of adverse outcomes predicted by the logistic regression models in Table 2. [file 13098_2022_940_MOESM1_ESM.docx]

**Additional file**

**What is an Appropriate Gestational Weight Gain for Women with Gestational Diabetes Mellitus: Based on the Adverse Pregnancy Outcomes of over 12 thousand Participants?**

Xiaoqin Luo^1¶^; Jiayi Gao^1¶^; Jing Ji^2^; Zhangya He^1^; Wanyu Zhang^1^; Pei Wu^1^; Xiaoxiao Guo^1^; Dan Cao^1^; Zhangrui Xu^1^; Chao Li^3^; Yang Mi^2*^

^1^ Department of Nutrition and Food Safety, School of Public Health, Xi’an Jiaotong University, Xi'an 710061, China

^2^ Department of Obstetrics and Gynecology, Northwest Women and Children’s Hospital, Xi’an 710061, China

^3^ Department of Epidemiology and Health Statistics, School of Public Health, Xi’an Jiaotong University, Xi’an 710061, China

^¶^The authors contribute equally to the paper.

*** Corresponding author**

Mi Yang, M.D.

Department of Obstetrics and Gynecology, Northwest Women’s and Children’s Hospital, Xi’an 710061, China

Telephone: +86-29-82655111 Fax: +86-29-82655111

E-mail: miyangmm@163.com

**Word count:** main text: 3293; abstract: 247

**Conflicts of interest.** The authors report no conflict of interest.

Singleton women with GDM

(n=14213)

Excluded records without height, weight, gestational weight gain (n=353)

Excluded preterm birth (n=820)

Excluded missing records (n=328)

Women with GDM eventually included in the study (n=12712)

Full-term singleton women with GDM (n=13065)

Included women who gave birth at NWCH from 2015 to 2018 (n=85211)

Excluded multiple births (n=3015)

Excluded non-GDM (n=67983)

**Figure S1 Flow chart of the participants**

**Table S1 Description of Missing value**

|  | N | Missing | |
| --- | --- | --- | --- |
|  |  | Count | Percent |
| HbA1c | 8934 | 3778 | 29.7 |
| Total cholesterol | 7360 | 5352 | 42.1 |
| Triglycerides | 7359 | 5353 | 42.1 |
| HDL | 7359 | 5353 | 42.1 |
| LDL | 7359 | 5353 | 42.1 |

**Table S2 *Chi*-square test for missing values**

|  | Pre-pregnancy Body Mass Index | | | | *P* value ^a^ |
| --- | --- | --- | --- | --- | --- |
|  | Underweight  (BMI<18.5) | Normal weight  (BMI18.5~24.9) | Overweight  (BMI25.0~29.9) | Obesity  (BMI≥30.0) |  |
| HbA1c |  |  |  |  | 0.218 |
| Missing | 348 (29.5) | 2747 (30.1) | 608 (29.0) | 75 (24.9) |  |
| Normal | 832 (70.5) | 6387 (69.9) | 1489 (71.0) | 226 (75.1) |  |
| Total cholesterol |  |  |  |  | 0.158 |
| Missing | 491 (41.6) | 3898 (42.7) | 845 (40.3) | 118 (39.2) |  |
| Normal | 689 (58.4) | 5236 (57.3) | 1252 (59.7) | 183 (60.8) |  |
| Triglycerides |  |  |  |  | 0.168 |
| Missing | 491 (41.6) | 3898 (42.7) | 846 (40.3) | 118 (39.2) |  |
| Normal | 689 (58.4) | 5236 (57.3) | 1251 (59.7) | 183 (60.8) |  |
| HDL |  |  |  |  | 0.168 |
| Missing | 491 (41.6) | 3898 (42.7) | 846 (40.3) | 118 (39.2) |  |
| Normal | 689 (58.4) | 5236 (57.3) | 1251 (59.7) | 183 (60.8) |  |
| LDL |  |  |  |  | 0.168 |
| Missing | 491 (41.6) | 3898 (42.7) | 846 (40.3) | 118 (39.2) |  |
| Normal | 689 (58.4) | 5236 (57.3) | 1251 (59.7) | 183 (60.8) |  |

^a^ Categorical variables are expressed as counts (percentages) and the chi-square test is used. P values <0.05 were considered statistically significant.

**Table S3 Description of outcomes by GWG category**

| GWG(kg) | N | Gestational hypertension | Preeclampsia | Cesarean section | LGA | SGA | adverse outcomes |
| --- | --- | --- | --- | --- | --- | --- | --- |
| **Underweight** | | | | | | | |
| 2.5~9.9 | 88 | 0 | 1(1.1) | 23(26.1) | 1(1.1) | 6(6.8) | 28(31.8) |
| 10~11.9 | 166 | 1(0.6) | 0 | 37(22.3) | 4(2.4) | 14(8.4) | 51(30.7) |
| 12~13.9 | 223 | 2(0.9) | 2(0.9) | 65(29.1) | 11(4.9) | 6(2.7) | 78(35.0) |
| 14~15.9 | 266 | 2(0.8) | 1(0.4) | 80(30.1) | 21(7.9) | 3(1.1) | 94(35.3) |
| 16~17.9 | 125 | 0 | 0 | 42(33.6) | 18(14.4) | 2(1.6) | 53(42.4) |
| 18~19.9 | 95 | 1(1.1) | 0 | 31(32.6) | 17(17.9) | 2(2.1) | 40(42.1) |
| 20~21.9 | 124 | 0 | 3(2.4) | 48(38.7) | 26(21.0) | 3(2.4) | 63(50.8) |
| ≥22 | 93 | 4(4.3) | 3(3.2) | 43(46.2) | 25(26.9) | 3(3.2) | 60(64.5) |
| **Normal weight** | | | | | | | |
| -4~3.9 | 70 | 1(1.4) | 1(1.4) | 26(37.1) | 4(5.7) | 7(10.0) | 32(45.7) |
| 4~5.9 | 155 | 1(0.6) | 1(0.6) | 70(45.2) | 13(8.4) | 8(5.2) | 81(52.3) |
| 6~7.9 | 375 | 2(0.5) | 4(1.1) | 170(45.3) | 39(10.4) | 18(4.8) | 200(53.3) |
| 8~9.9 | 569 | 8(1.4) | 2(0.4) | 234(41.1) | 67(11.8) | 20(3.5) | 277(48.7) |
| 10~11.9 | 1624 | 26(1.6) | 19(1.2) | 708(43.6) | 246(15.1) | 49(3.0) | 870(53.6) |
| 12~13.9 | 1506 | 25(1.7) | 23(1.5) | 673(44.7) | 286(19.0) | 29(1.9) | 830(55.1) |
| 14~15.9 | 2002 | 41(2.0) | 37(1.8) | 909(45.4) | 420(21.0) | 55(2.7) | 1144(57.1) |
| 16~17.9 | 1026 | 9(0.9) | 26(2.5) | 448(43.7) | 252(24.6) | 19(1.9) | 580(56.5) |
| 18~19.9 | 482 | 5(1.0) | 12(2.5) | 233(48.3) | 119(24.7) | 3(0.6) | 283(58.7) |
| 20~21.9 | 852 | 15(1.8) | 30(3.5) | 405(47.5) | 264(31.0) | 9(1.1) | 533(62.6) |
| 22~23.9 | 177 | 4(2.3) | 7(4.0) | 95(53.7) | 50(28.2) | 2(1.1) | 113(63.8) |
| 24~25.9 | 210 | 8(3.8) | 8(3.8) | 105(50.0) | 87(41.4) | 3(1.4) | 147(70.0) |
| ≥26 | 86 | 4(4.7) | 3(3.5) | 52(60.5) | 42(48.8) | 2(2.3) | 70(81.4) |
| **Overweight** | | | | | | | |
| -8~3.9 | 78 | 0 | 3(3.8) | 46(59.0) | 12(15.4) | 3(3.8) | 52(66.7) |
| 4~5.9 | 123 | 4(3.3) | 4(3.3) | 74(60.2) | 20(16.3) | 4(3.3) | 86(69.9) |
| 6~7.9 | 174 | 3(1.7) | 7(4.0) | 92(52.9) | 43(24.7) | 6(3.4) | 117(67.2) |
| 8~9.9 | 177 | 6(3.4) | 3(1.7) | 101(57.1) | 39(22.0) | 4(2.3) | 122(68.9) |
| 10~11.9 | 483 | 27(5.6) | 26(5.4) | 294(60.9) | 138(28.6) | 10(2.1) | 345(71.4) |
| 12~13.9 | 315 | 12(3.8) | 6(1.9) | 190(60.3) | 98(31.1) | 3(1.0) | 231(73.3) |
| 14~15.9 | 379 | 16(4.2) | 24(6.3) | 227(59.9) | 156(41.2) | 3(0.8) | 285(75.2) |
| 16~17.9 | 129 | 7(5.4) | 6(4.7) | 71(55.0) | 41(31.8) | 2(1.6) | 91(70.5) |
| 18~19.9 | 50 | 2(4.0) | 5(10.0) | 33(66.0) | 19(38.0) | 2(4.0) | 39(78.0) |
| 20~21.9 | 122 | 9(7.4) | 12(9.8) | 82(67.2) | 52(42.6) | 1(0.8) | 100(82.0) |
| ≥22 | 67 | 5(7.5) | 11(16.4) | 47(70.1) | 27(40.3) | 2(3.0) | 53(79.1) |
| **Obesity** | | | | | | | |
| -5~3.9 | 26 | 1(3.8) | 2(7.7) | 12(46.2) | 7(26.9) | 1(3.8) | 18(69.2) |
| 4~5.9 | 28 | 2(7.1) | 0 | 20(71.4) | 10(35.7) | 0 | 22(78.6) |
| 6~7.9 | 36 | 4(11.1) | 1(2.8) | 28(77.8) | 7(19.4) | 2(5.6) | 31(86.1) |
| 8~9.9 | 42 | 1(2.4) | 7(16.7) | 30(71.4) | 16(38.1) | 1(2.4) | 37(88.1) |
| 10~11.9 | 62 | 7(11.3) | 9(14.5) | 36(58.1) | 22(35.5) | 0 | 44(71.0) |
| 12~13.9 | 30 | 1(3.3) | 4(13.3) | 18(60.0) | 11(36.7) | 0 | 22(73.3) |
| ≥14 | 77 | 15(19.5) | 13(16.9) | 60(77.9) | 37(48.1) | 0 | 67(87.0) |

**Table S4 Univariate logistic regression analysis of continuous GWG and pre-pregnancy BMI with adverse outcomes**

| Characteristics | OR (95%CI) | P value |
| --- | --- | --- |
| pre-pregnancy BMI | 1.153 (1.139~1.167) | <0.001 |
| GWG | 1.021 (1.014~1.029) | <0.001 |

**Table S5 Associations of GWG categories with adverse outcomes in the Underweight group***

| GWG(kg) | N | Model 1^a^ | | Model 2^b^ | | Model 3^c^ | | Model 4^d^ | |
| --- | --- | --- | --- | --- | --- | --- | --- | --- | --- |
|  |  | P value | OR (95%CI) | P value | OR (95%CI) | P value | OR (95%CI) | P value | OR (95%CI) |
| 2.5~9.9 | 88 | 0.233 | 0.747  (0.463~1.206) | 0.257 | 0.745  (0.448~1.239) | 0.127 | 0.687  (0.424~1.113) | 0.222 | 0.726  (0.434~1.214) |
| 10.0~11.9 | 166 | 0.027 | 0.662  (0.459~0.953) | 0.067 | 0.697  (0.473~1.026) | 0.018 | 0.643  (0.446~0.927) | 0.061 | 0.691  (0.469~1.017) |
| 12.0~13.9 | 223 | 0.072 | 0.748  (0.545~1.026) | 0.075 | 0.730  (0.516~1.032) | 0.041 | 0.718  (0.522~0.987) | 0.065 | 0.721  (0.509~1.021) |
| 14.0~15.9 | 266 | 0.030 | 0.719  (0.534~0.968) | 0.030 | 0.696  (0.502~0.965) | 0.029 | 0.718  (0.533~0.968) | 0.030 | 0.696  (0.502~0.966) |
| 16.0~17.9 | 125 | 0.364 | 1.197  (0.811~1.767) | 0.853 | 1.041  (0.679~1.597) | 0.240 | 1.266  (0.854~1.876) | 0.798 | 1.058  (0.688~1.627) |
| 18.0~19.9 | 95 | 0.715 | 1.085  (0.699~1.686) | 0.613 | 1.129  (0.705~1.808) | 0.674 | 1.100  (0.706~1.713) | 0.601 | 1.134  (0.708~1.816) |
| 20.0~21.9 | 124 | 0.006 | 1.716  (1.165~2.528) | 0.002 | 1.865  (1.247~2.788) | 0.003 | 1.814  (1.226~2.686) | 0.002 | 1.896  (1.266~2.841) |
| ≥22.0 | 93 | <0.001 | 3.427  (2.171~5.407) | <0.001 | 3.414  (2.139~5.449) | 0.000 | 3.781  (2.379~6.009) | <0.001 | 3.526  (2.200~5.649) |

*The reference for each GWG category was the GWG beyond the range.

^a^ Model 1 were adjusted for age and parity.

^b^ Model 2 were adjusted for age, parity and previous cesarean section history.

^c^ Model 3 were adjusted for age, parity and gestational week.

^d^ Model 4 were adjusted for age, parity, gestational week and previous cesarean section history.

**Table S6 Associations of GWG categories with adverse outcomes in Normal weight group***

| GWG(kg) | N | Model 1^a^ | | Model 2^b^ | | Model 3^c^ | | Model 4^d^ | |
| --- | --- | --- | --- | --- | --- | --- | --- | --- | --- |
|  |  | P value | OR (95%CI) | P value | OR (95%CI) | P value | OR (95%CI) | P value | OR (95%CI) |
| -4~3.9 | 70 | 0.030 | 0.586  (0.362~0.950) | 0.049 | 0.586  (0.344~0.999) | 0.012 | 0.537  (0.331~0.872) | 0.032 | 0.558  (0.327~0.952) |
| 4~5.9 | 155 | 0.162 | 0.793  (0.573~1.097) | 0.190 | 0.788  (0.551~1.126) | 0.057 | 0.727  (0.524~1.009) | 0.111 | 0.747  (0.522~1.070) |
| 6~7.9 | 375 | 0.087 | 0.831  (0.673~1.027) | 0.041 | 0.782  (0.619~0.989) | 0.020 | 0.776  (0.626~0.961) | 0.018 | 0.752  (0.594~0.952) |
| 8~9.9 | 569 | 0.000 | 0.643  (0.540~0.766) | <0.001 | 0.690  (0.570~0.835) | <0.001 | 0.628  (0.527~0.748) | <0.001 | 0.678  (0.559~0.821) |
| 10~11.9 | 1624 | 0.000 | 0.806  (0.722~0.900) | <0.001 | 0.798  (0.707~0.901) | <0.001 | 0.793  (0.709~0.886) | <0.001 | 0.791  (0.701~0.894) |
| 12~13.9 | 1506 | 0.131 | 0.916  (0.818~1.026) | 0.157 | 0.915  (0.809~1.035) | 0.114 | 0.912  (0.813~1.023) | 0.145 | 0.913  (0.807~1.032) |
| 14~15.9 | 2002 | 0.426 | 1.042  (0.941~1.154) | 0.664 | 1.025  (0.918~1.144) | 0.434 | 1.042  (0.940~1.155) | 0.660 | 1.025  (0.918~1.145) |
| 16~17.9 | 1026 | 0.540 | 1.043  (0.912~1.191) | 0.842 | 0.985  (0.853~1.138) | 0.345 | 1.067  (0.933~1.221) | 0.992 | 0.999  (0.865~1.154) |
| 18~19.9 | 482 | 0.122 | 1.161  (0.961~1.403) | 0.032 | 1.244  (1.020~1.518) | 0.096 | 1.176  (0.972~1.424) | 0.029 | 1.249  (1.023~1.524) |
| 20~21.9 | 852 | 0.000 | 1.448  (1.249~1.678) | <0.001 | 1.462  (1.251~1.708) | <0.001 | 1.486  (1.280~1.726) | <0.001 | 1.485  (1.270~1.736) |
| 22~23.9 | 177 | 0.011 | 1.504  (1.098~2.059) | 0.071 | 1.358  (0.974~1.894) | 0.003 | 1.624  (1.180~2.234) | 0.039 | 1.422  (1.019~1.984) |
| 24~25.9 | 210 | <0.001 | 2.112  (1.562~2.854) | <0.001 | 2.152  (1.578~2.934) | <0.001 | 2.323  (1.714~3.149) | <0.001 | 2.261  (1.657~3.086) |
| ≥26 | 86 | <0.001 | 3.869  (2.233~6.702) | <0.001 | 4.138  (2.377~7.203) | <0.001 | 4.180  (2.405~7.265) | <0.001 | 4.272  (2.452~7.442) |

*The reference for each GWG category was the GWG beyond the range.

^a^ Model 1 were adjusted for age and parity.

^b^ Model 2 were adjusted for age, parity and previous cesarean section history.

^c^ Model 3 were adjusted for age, parity and gestational week.

^d^ Model 4 were adjusted for age, parity, gestational week and previous cesarean section history.

**Table S7 Associations of GWG categories with adverse outcomes in Overweight group***

| GWG(kg) | N | Model 1^a^ | | Model 2^b^ | | Model 3^c^ | | Model 4^d^ | |
| --- | --- | --- | --- | --- | --- | --- | --- | --- | --- |
|  |  | P value | OR (95%CI) | P value | OR (95%CI) | P value | OR (95%CI) | P value | OR (95%CI) |
| -8~3.9 | 78 | 0.057 | 0.618  (0.377~1.014) | 0.018 | 0.507  (0.289~0.892) | 0.044 | 0.598  (0.362~0.986) | 0.019 | 0.507  (0.287~0.896) |
| 4~5.9 | 123 | 0.312 | 0.811  (0.540~1.217) | 0.179 | 0.739  (0.475~1.149) | 0.190 | 0.759  (0.503~1.146) | 0.115 | 0.700  (0.449~1.091) |
| 6~7.9 | 174 | 0.044 | 0.705  (0.502~0.991) | 0.087 | 0.729  (0.507~1.047) | 0.035 | 0.689  (0.488~0.974) | 0.069 | 0.713  (0.495~1.026) |
| 8~9.9 | 177 | 0.183 | 0.793  (0.564~1.116) | 0.210 | 0.793  (0.552~1.139) | 0.197 | 0.797  (0.564~1.126) | 0.225 | 0.798  (0.554~1.149) |
| 10~11.9 | 483 | 0.429 | 0.911  (0.724~1.147) | 0.396 | 0.900  (0.724~1.147) | 0.385 | 0.902  (0.715~1.138) | 0.387 | 0.898  (0.703~1.146) |
| 12~13.9 | 315 | 0.997 | 0.999  (0.758~1.317) | 0.738 | 0.951  (0.709~1.276) | 0.944 | 0.990  (0.749~1.309) | 0.725 | 0.948  (0.706~1.274) |
| 14~15.9 | 379 | 0.108 | 1.238  (0.954~1.605) | 0.050 | 1.310  (1.000~1.717) | 0.078 | 1.267  (0.974~1.649) | 0.045 | 1.320  (1.006~1.732) |
| 16~17.9 | 129 | 0.815 | 0.954  (0.641~1.420) | 0.857 | 0.962  (0.634~1.461) | 0.855 | 0.963  (0.644~1.440) | 0.893 | 0.972  (0.638~1.479) |
| 18~19.9 | 50 | 0.276 | 1.463  (0.738~2.900) | 0.390 | 1.363  (0.673~2.759) | 0.231 | 1.526  (0.764~3.049) | 0.354 | 1.398  (0.688~2.840) |
| 20~21.9 | 122 | 0.002 | 2.094  (1.299~3.376) | 0.001 | 2.260  (1.393~3.668) | 0.001 | 2.266  (1.395~3.679) | 0.001 | 2.357  (1.448~3.837) |
| ≥22 | 67 | 0.056 | 1.801  (0.985~3.295) | 0.086 | 1.711  (0.927~3.155) | 0.061 | 1.795  (0.974~3.310) | 0.087 | 1.712  (0.925~3.168) |

*The reference for each GWG category was the GWG beyond the range.

^a^ Model 1 were adjusted for age and parity.

^b^ Model 2 were adjusted for age, parity and previous cesarean section history.

^c^ Model 3 were adjusted for age, parity and gestational week.

^d^ Model 4 were adjusted for age, parity, gestational week and previous cesarean section history.

**Table S8 Associations of** **GWG categories with adverse outcomes in Obesity group***

| GWG(kg) | N | Model 1^a^ | | Model 2^b^ | | Model 3^c^ | | Model 4^d^ | |
| --- | --- | --- | --- | --- | --- | --- | --- | --- | --- |
|  |  | P value | OR (95%CI) | P value | OR (95%CI) | P value | OR (95%CI) | P value | OR (95%CI) |
| -5~3.9 | 26 | 0.105 | 0.473  (0.191~1.169) | 0.041 | 0.360  (0.135~0.961) | 0.127 | 0.474  (0.182~1.236) | 0.066 | 0.386  (0.140~1.065) |
| 4~5.9 | 28 | 0.950 | 1.031  (0.395~2.692) | 0.793 | 0.872  (0.313~2.428) | 0.775 | 1.154  (0.433~3.076) | 0.992 | 1.005  (0.352~2.870) |
| 6~7.9 | 36 | 0.305 | 1.687  (0.622~4.576) | 0.440 | 1.524  (0.523~4.439) | 0.380 | 1.588  (0.566~4.455) | 0.559 | 1.387  (0.463~4.157) |
| 8~9.9 | 42 | 0.192 | 1.929  (0.720~5.168) | 0.192 | 1.970  (0.712~5.451) | 0.151 | 2.105  (0.761~5.819) | 0.150 | 2.151  (0.758~6.099) |
| 10~11.9 | 62 | 0.038 | 0.503  (0.263~0.962) | 0.108 | 0.570  (0.287~1.132) | 0.009 | 0.394  (0.196~0.793) | 0.028 | 0.440  (0.211~0.916) |
| 12~13.9 | 30 | 0.395 | 0.686  (0.287~1.638) | 0.519 | 0.738  (0.294~1.856) | 0.720 | 0.847  (0.342~2.099) | 0.854 | 0.914  (0.350~2.383) |
| ≥14 | 77 | 0.089 | 1.900  (0.907~3.980) | 0.065 | 2.051  (0.955~4.405) | 0.098 | 1.919  (0.887~4.152) | 0.077 | 2.027  (0.927~4.429) |

*The reference for each GWG category was the GWG beyond the range.

^a^ Model 1 were adjusted for age and parity.

^b^ Model 2 were adjusted for age, parity and previous cesarean section history.

^c^ Model 3 were adjusted for age, parity and gestational week.

^d^ Model 4 were adjusted for age, parity, gestational week and previous cesarean section history.

**Table S9 The results of adverse outcomes predicted by the logistic regression models in Table 2**

| NRI calculation table ^a^ | | | |
| --- | --- | --- | --- |
|  | This study | | Total |
|  | Yes^b^ | No^c^ |  |
| adverse outcomes occurred | | | |
| IOM |  |  |  |
| Yes^d^ | 5325(a1) | 385(b1) | 5710 |
| No^e^ | 27(c1) | 1652(d1) | 1679 |
| Total | 5352 | 2037 | 7389(N1) |
| No adverse outcomes occurred | | | |
| IOM |  |  |  |
| Yes^d^ | 2847(a2) | 632(b2) | 3479 |
| No^e^ | 27(c2) | 1817(d2) | 1844 |
| Total | 2874 | 2449 | 5323(N2) |

^a^ NRI=(c1-b1)/N1+(b2-c2)/N2=0.065>0, Z=$\frac{NRI}{\sqrt{\frac{b1+c1}{{N1}^{2}}+\frac{b2+c2}{{N2}^{2}}}}$=11.71，*P* value < 0.001. NRI is statistically significant compared with 0, which shows that the predictive ability of This study is better than IOM.

^b^ In the model of this study, it is predicted that adverse outcomes will occur.

^c^ In the model of this study, it is predicted that adverse outcomes will not occur.

^d^ In the model of IOM, it is predicted that adverse outcomes will occur.

^e^ In the model of IOM, it is predicted that adverse outcomes will not occur.
